# A Nondestructive Methodology for Determining Chemical Composition of *Salvia miltiorrhiza* via Hyperspectral Imaging Analysis and Squeeze-and-Excitation Residual Networks

**DOI:** 10.3390/s23239345

**Published:** 2023-11-23

**Authors:** Jieqiang Zhu, Jiaqi Bao, Yi Tao

**Affiliations:** College of Pharmaceutical Science, Zhejiang University of Technology, Hangzhou 310032, China; zhujieqiang@zjut.edu.cn (J.Z.); 2112107165@zjut.edu.cn (J.B.)

**Keywords:** hyperspectral image, *Salvia miltiorrhiza*, squeeze-and-excitation residual network, process analysis technology, intelligent process analysis

## Abstract

The quality assurance of bulk medicinal materials, crucial for botanical drug production, necessitates advanced analytical methods. Conventional techniques, including high-performance liquid chromatography, require extensive pre-processing and rely on extensive solvent use, presenting both environmental and safety concerns. Accordingly, a non-destructive, expedited approach for assessing both the chemical and physical attributes of these materials is imperative for streamlined manufacturing. We introduce an innovative method, designated as Squeeze-and-Excitation Residual Network Combined Hyperspectral Image Analysis (SE-ReHIA), for the swift and non-invasive assessment of the chemical makeup of bulk medicinal substances. In a demonstrative application, hyperspectral imaging in the 389–1020 nm range was employed in 187 batches of *Salvia miltiorrhiza*. Notable constituents such as salvianolic acid B, dihydrotanshinone I, cryptotanshinone, tanshinone IIA, and moisture were quantified. The SE-ReHIA model, incorporating convolutional layers, maxpooling layers, squeeze-and-excitation residual blocks, and fully connected layers, exhibited $R_{c}^{2}$ values of 0.981, 0.980, 0.975, 0.972, and 0.970 for the aforementioned compounds and moisture. Furthermore, $R_{p}^{2}$ values were ascertained to be 0.975, 0.943, 0.962, 0.957, and 0.930, respectively, signifying the model’s commendable predictive competence. This study marks the inaugural application of SE-ReHIA for Salvia miltiorrhiza’s chemical profiling, offering a method that is rapid, eco-friendly, and non-invasive. Such advancements can fortify consistency across botanical drug batches, underpinning product reliability. The broader applicability of the SE-ReHIA technique in the quality assurance of bulk medicinal entities is anticipated with optimism.

## 1. Introduction

The efficacy of Chinese patent drugs hinges significantly on the integrity of the raw materials, predominantly bulk medicinal materials, employed in their formulation. Ensuring rigorous quality control of these raw materials is paramount to ascertain the reliability of the final products. Though high-performance liquid chromatography (HPLC) has been acknowledged for its routine utility in quality assessment, its limitations cannot be overlooked. Notably, preliminary sample pretreatment before HPLC is time-intensive, and the HPLC analytical process mandates the use of considerable volumes of potentially hazardous organic solvents, including acetonitrile and methanol, challenging the principles of green chemistry. Advancements in process analytical technology (PAT) proffer alternative methodologies for evaluating the quality metrics of bulk medicinal materials.

Of these, hyperspectral image analysis (HSI) emerges as a novel PAT instrument gaining traction amongst pharmaceutical researchers. The potential of HSI in medicinal material identification has been demonstrated; for instance, Sandasi et al. discerned three analogous Echinacea species employing HSI in conjunction with chemometric classification modeling, thereby achieving accurate identification in commercial formulations [1]. Furthermore, Vermaak et al. presented an expedited strategy to differentiate I.anisatum and I.verum dried fruits, employing HSI and analytical techniques such as principal component analysis and partial least squares discriminant analysis [2]. While there are instances of quantitative HSI applications, such as in predicting free fatty acid concentrations in stored chia seeds using near-infrared HSI combined with chemometrics [3] and in determining the composition of herbal tea mixtures [4], investigations focusing on quantitative applications of HSI remain sparse.

Distinct from conventional images, the hyperspectrum emanating from hyperspectral instruments represents a data cube. This not only encapsulates the essence of the image but also extends into the spectral dimension. Conventional extraction methods pertinent to panchromatic or multispectral imaging are not congruent with hyperspectral image processing. In a noteworthy endeavor, Huang et al. introduced a hyperspectral imaging technique coupled with a one-dimensional convolutional neural network (1D-CNN) model, achieving near-perfect precision and sensitivity [5]. Furthermore, Rodrige et al. presented an innovative sliding window variographic image analysis technique [6], underlining the imperative to conceive novel information extraction paradigms tailored to the unique properties of hyperspectral images.

Herein, we introduce an avant-garde approach, termed squeeze-and-excitation residual network combined hyperspectral image analysis (SE-ReHIA), devised for the non-invasive and swift determination of quality markers in bulk medicinal materials. The underpinning of the SE-ReHIA method rests on the melding of residual networks (ResNets) and squeeze-and-excitation networks (SE-Nets), both of which epitomize recent advancements in image recognition. ResNets tactically tackle the vanishing gradient issue by incorporating direct shortcut connections, thus circumventing potential impediments of intermediate layers [7]. Conversely, SE-Nets hone in on accentuating salient features while concurrently downplaying non-essential ones through the judicious use of global pooling succeeded by adaptive weights [8,9]. Squeeze-and-excitation (SE) blocks were successfully utilized for diabetic retinopathy (DR)-related biomarker detection [10] and android malware detection [11].

Exemplifying this methodology, *Salvia miltiorrhiza* (SM)—lauded for its multifarious therapeutic benefits including cardiovascular protection [12,13], anti-inflammatory [14], antitumor [15], antioxidative [16], antifibrotic [17], and antidiabetic properties [18]—was chosen. Hyperspectral images from 187 SM batches were sourced via a hyperspectral imaging apparatus. In adherence with the Chinese Pharmacopoeia (CHP) guidelines, SM’s moisture content could not breach the 13.0% threshold, with specified contents of key compounds [19]. Using HPLC-DAD and a rapid moisture analyzer, we ascertained the content profiles of these pivotal compounds across SM batches. Consequently, a quantitative calibration model rooted in SE-ReHIA was sculpted to deduce the content of the quintessential components. To rigorously benchmark SE-ReHIA, alternative models including partial least squares regression (PLSR), support vector machine regression (SVMR), and radial basis function neural networks (RBFNN) were also created.

The novelty of the SE-ReHIA method lies in the following three points. First, the SE-ReHIA method was initially proposed for the nondestructive and rapid determining of the chemical composition of Salvia Miltiorrhizae. Second, the predictive ability of SE-ResNet is superior to that of PLSR, SVMR and RBFNN. Third, the Rp^2^ values of the five quality attributes are all above 0.9300.

## 2. Materials and Methods

### 2.1. Chemicals and Reagents

Standardized compounds, encompassing salvianolic acid B, dihydrotanshinone I, cryptotanshinone, and tanshinone IIA, were procured from Sichuan Weikeqi Biological Technology Co. (Sichuan, China). All aqueous solutions were prepared utilizing distilled water from a Milli-Q Reagent Water System (Millipore, MA, USA).

A generous donation from Zhengda Qingchunbao Co. (Zhejiang, China) provided eight batches of SM samples. In addition to this, SM samples from diverse regions were acquired: Sichuan Province (4 batches), Yunnan Province (4 batches), Shanxi Province (22 batches), Anhui Province (23 batches), Henan Province (58 batches), and Shandong Province (68 batches). An exhaustive list of the 187 SM batches is presented in Table S1. All samples underwent rigorous authentication under the expert guidance of Prof. Ping Wang, Zhejiang University of Technology. Corresponding voucher specimens have been curated and securely archived in the herbarium of the College of Pharmaceutical Sciences at the Zhejiang University of Technology.

### 2.2. Hyperspectral Images Acquisition

**Figure 1:**
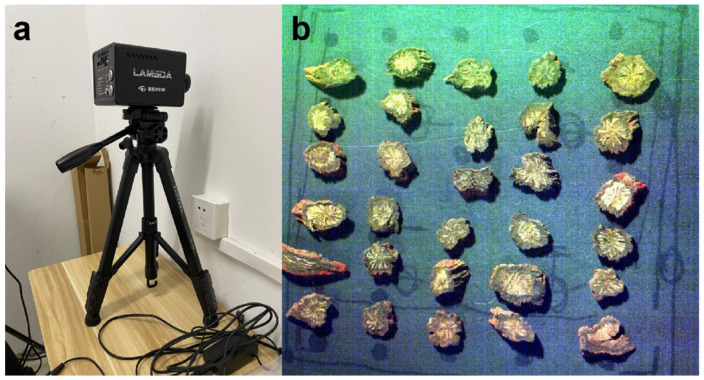
Hyperspectral images system (**a**) and samples of *Salvia miltiorrhiza* (**b**).

For each acquired batch, segments of *Salvia miltiorrhiza* were methodically positioned in a matrix configuration on a Teflon plate, adhering to a pattern of 6 segments per row and 5 segments per column, as depicted in Figure 1.

The imaging process employed a Lambda-Nir hyperspectral camera (Wuxi Spectrum Vision Technology Co., Wuxi, China), capturing at intervals of precisely 5.38 nm within the visible and near-infrared spectrum, ranging from 380 nm to 1064 nm. This spanned a total of 128 distinct bands and operated at a spectral resolution of 10 nm. In an endeavor to preserve the fidelity of the captured images, dimensions were meticulously set at 800 pixels in width by 703 pixels in height. Subsequent empirical evaluations ascertained that an optimal camera configuration comprised an exposure duration of 2.3 ms and a designated 40 cm gap between the camera lens and the sample substrate. Utilizing these optimized settings, high-quality hyperspectral images were acquired for all 187 batches of *Salvia miltiorrhiza*.

### 2.3. Hyperspectral Image Correction

In order to counteract the potential perturbations introduced by dark currents, uneven light distribution, and the extended operation of heat-generating instruments, a standardized whiteboard calibration procedure was employed. Specifically, an image of a calibration whiteboard was captured for reference. Simultaneously, a calibration image was procured with the camera lens cover in place, providing a blackboard calibration counterpart. These calibration images were subsequently integrated into the HSI system’s intrinsic image acquisition software, ensuring the accurate calibration of reflectivity across the spectrum of acquired hyperspectral images.

### 2.4. HPLC Analysis

All *Salvia miltiorrhiza* (SM) batches underwent pulverization using a specialized Chinese medicine pulverizer, and were subsequently sieved through a 50-mesh filter. An exact weight of 0.5 g of the resultant powdered sample was meticulously combined with 25 mL of a mixed solvent, characterized by an 80:20 (*v*/*v*) ratio of methanol to water. This mixture was subjected to ultrasonic extraction for a duration of 40 min. Post-extraction, the solution was centrifuged at a speed of 13,000 rpm for 5 min. The ensuing supernatant, after filtration through a 0.22 μm membrane, was readied for HPLC injection.

HPLC analysis was conducted using the Agilent 1260 HPLC system (Agilent Technologies, California, USA), a comprehensive system encompassing a binary pump, a sample vial injector, a column oven, and a diode array detector (DAD). The chromatographic separation was performed on a Waters XBridge C_18_ column (4.6 × 250 mm, 5 μm) maintained at a temperature of 35 °C. The employed mobile phases comprised (A) 0.1% formic acid in water (HCOOH-H_2_O) and (B) acetonitrile. The linear gradient elution was methodically structured: 0–15 min with a transition from 90% to 60% of (A); 15–19 min adjusting from 60% to 36% of (A); and finally, 19–32 min transitioning from 36% to 10% of (A). The system operated at a flow rate of 1.0 mL/min. The detection wavelength for the compounds salvianolic acid B, dihydrotanshinone I, cryptotanshinone, and tanshinone IIA was uniformly set at 288 nm.

### 2.5. Method Validation

Precise amounts of salvianolic acid B, dihydrotanshinone I, cryptotanshinone, and tanshinone IIA, each weighing 1 mg, were separately solubilized in methanol to generate standard stock solutions. Subsequent dilutions of these stock solutions yielded working solutions at specified concentrations. The linearity criterion, indicative of the proportionality between a compound’s peak area and its concentration over the stipulated range, necessitates a correlation coefficient (R^2^) of no less than 0.9990. Analytical signals for the quartet of compounds exhibited intensities approximately thrice that of the baseline noise at the limit of detection (LOD) and a magnitude about tenfold at the limit of quantitation (LOQ). Intra-day precision was ascertained through sextuple samplings over a single day, whereas inter-day precision was evaluated through tripartite samplings over three sequential days. To assess reproducibility, a parallel setup of six samples was established for uninterrupted injection analysis. Time-based stability analysis of the samples was performed at intervals of 0, 2, 4, 8, 16, and 24 h. The method’s recovery rate was determined utilizing the standard addition method, with the recovery percentage calculated using the formula: Recovery (%) = [(amount identified − initial amount)/amount augmented] × 100%.

### 2.6. Moisture Determination

A swift analytical method for quantifying moisture content in SM was developed. Each batch of SM was subjected to milling processes to achieve a powdered consistency, followed by sieving through a 20-mesh standard. An aliquot of this SM powder was assessed for its moisture content to serve as a reference, adhering to the specifications laid out by the second method of moisture determination as indicated in CHP [18]. Subsequently, an exhaustive set of factorial experiments were conducted to optimize the parameters of the rapid moisture analyzer. The established conditions comprised a heating temperature of 105 °C, a sample mass of 3 g, and a discrimination time of 40 s. Operating under these conditions, moisture content was ascertained for 187 distinct SM batches. For each batch, duplicate measurements were taken, with the average of the two serving as the definitive moisture content.

### 2.7. Establishment of PLSR Model

In an effort to evaluate the predictive accuracy of the refined SE-ResNet model, a PLSR calibration model was established for the quantification of the same analytes. Within the framework of the PLSR model, various spectral preprocessing techniques, alongside feature band filtering algorithms, were investigated. The preprocessing methodologies assessed encompassed Savitzky–Golay smoothing and the first derivative. Meanwhile, the feature band filtering methodologies explored included competitive adaptive reweighted sampling (CARS), the successive projections algorithm, and the uninformative variable elimination technique.

### 2.8. Establishment of SVMR and RBFNN Models

Support vector machine regression (SVMR) was conducted in high-dimensional space by using the Vapink loss function, which consists of empirical error and regularization terms. SVR was applied to the average spectral data and five chemical composition values. The prediction function was trained to predict the five chemical composition values of the sample, where the average spectral data of the *i*th sample represented *j*th chemical composition values of the *i*th sample.

In the architectural domain of radial basis function neural networks (RBFNN), a trilayered structure is evident: an introductory layer, a concealed intermediary layer, and a conclusive output layer. The primary role of the introductory layer is to facilitate the propagation of input vectors towards the intermediary hidden layer. This concealed layer is fundamentally composed of an array of radial basis function units, represented as bk. Each constituent of this hidden layer exemplifies an individual radial basis function, equipped with a distinct center position and delineated width. Intriguingly, the input data set undergoes a transformation mediated by the Gaussian function, intrinsically defined by its center cj and breadth rj. Such a radial basis function (RBF) is instrumental in computing the Euclidean distance between a given input vector (x) and the respective center of the radial basis function (cj).

### 2.9. Establishment of SE-ResNet Model

For building a quantitative calibration model for the contents of four active compounds and moisture, the SE-ResNet algorithm was applied. An SE block is a computational unit which can be built upon a transformation Ftr mapping an input X ϵ ℝ^H’×W’×C’^ to feature maps U ϵ ℝ^H×W×C^. Taking Ftr to be a convolutional operator and using V = [v_1_, v_2_, …, v_c_] to denote the learned set of filter kernels, where vc refers to the parameters of the c-th filter. Then the outputs as U = [u_1_, u_2_, …, u_c_],

(1) $$u_{c}=v_{c}\times X=\sum_{s=1}^{c^{\prime }}v_{c}^{s}\times x^{s}$$

where here × denotes convolution, $v_{c}$ = [$v_{c}^{1}$, $v_{c}^{2}$, …, $v_{c}^{c\prime }$], X = [x_1_, x_2_, …, x_c’_] and uc ϵ ℝ^H×W^. $v_{c}^{s}$ is a 2D spatial kernel representing a single channel of vc that acts on the corresponding channel of X.

**Figure 2:**
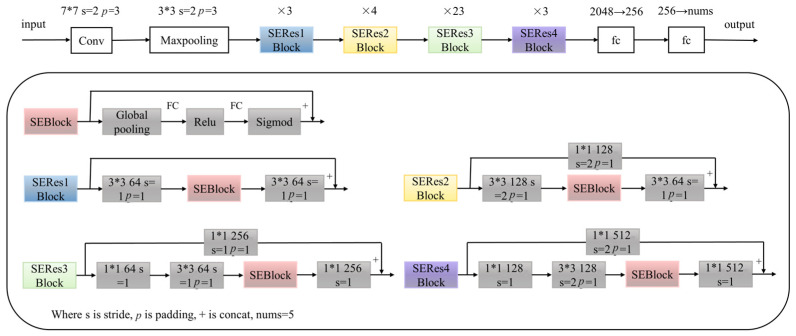
The architecture of the SE-ResNet model.

The schematic representation of the SE-ResNet model under consideration can be found in Figure 2. This model comprises various components, starting with an input layer followed by a convolutional layer and a subsequent batch normalization layer. In the convolutional structure of this model, distinct SE-ResBlocks are utilized: thrice for SE-Res1Block, fourfold for SE-Res2Block, twenty-three times for SE-Res3Block, and thrice for SE-Res4Block. The initial convolutional layer that the hyperspectral data encounters is characterized by hyperparameters: a filter window dimension of 7 × 7, a stride of 2, and a padding value of 3. Post this, the data are directed to a maxpooling layer, with convolution parameters being a filter window of 3 × 3, stride of 2, and padding value of 3. Subsequently, the data transit through two fully connected layers. On entry to the primary fully connected layer, there is a reduction in neuron count from 2048 to 256, culminating in an output neuron count of 5 in the subsequent fully connected layer.

### 2.10. Assessment of the Established Models

All models were created for regression analysis, and the performance of the established models was evaluated by the calculation of the root mean square error (RMSE) and correlation coefficient according to Equations (2) and (3). They can be divided into root mean square error of calibration (RMSEC), root mean square error of cross-validation (RMSECV), the root mean square error of prediction (RMSEP), correlation coefficient of calibration ($R_{c}^{2}$), correlation coefficient of cross-validation ($R_{cv}^{2}$), and correlation coefficient of prediction ($R_{p}^{2}$).

(2) $$RMSE=\sqrt{\frac{\sum_{i=1}^{N}{\left(\hat{c_{i}}-c_{i}\right)}^{2}}{n}}$$

(3) $$R^{2}=1-\frac{\sum_{i=1}^{N}{\left(\hat{c_{i}}-c_{i}\right)}^{2}}{\sum_{i=1}^{N}{\left(\hat{c_{i}}-\bar{c_{i}}\right)}^{2}}$$

where $c_{i}$ is the actual result for sample i, $\hat{c_{i}}$ is the estimated value by model for the sample i, n is the number of samples, and $\bar{c_{i}}$ is the mean of the actual results for samples.

The accuracy of the calibration model was evaluated by $R_{c}^{2}$, $R_{cv}^{2}$, and $R_{p}^{2}$, whereas the precision of the model was assessed using RMSEC, RMSECV and RMSEP. Additionally, the residual prediction deviation (RPD) and relative error range (RER) were calculated to evaluate the reliability, robustness, and predictive capability of the regression models. RPD was calculated according to Equation (4). RER was defined in Equation (5).

(4) $$RPD=\frac{{DP}_{cal}}{RMSEP}$$

(5) $$RER=\frac{Y_{max}-Y_{min}}{RMSEP}$$

where ${DP}_{cal}$ is the standard deviation of the calibration set, $Y_{max}$ is the maximum value of quality attributes, and $Y_{min}$ is the minimum value of quality attributes.

An RPD value below 1.5 suggests limited utility of the model. A range of 1.5 < RPD < 2.0 is indicative of the model’s capability to discriminate between high and low values. RPD values falling within 2.0 and 2.5 suggest an approximate predictive potential. A range between 2.5 and 3.0 is demonstrative of the model’s commendable predictive proficiency, while an RPD exceeding 3 is emblematic of superior predictive performance. Additionally, larger RER values are directly proportional to enhanced predictive capacity.

## 3. Results

### 3.1. Quantitation of Effective Ingredients

**Figure 3:**
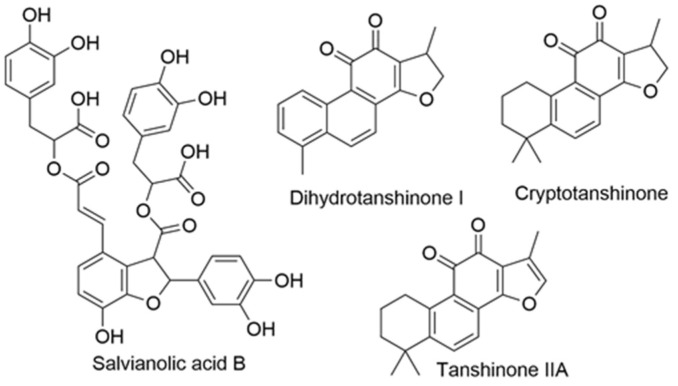
The chemical structures of four investigated analytes.

The reliability and precision of the HPLC-DAD method in determining the content of the aforementioned active compounds in *Salvia miltiorrhiza* (SM) samples is unequivocally substantiated by the analysis of 187 distinct batches. The intrinsic UV absorption characteristics of these compounds make them readily detectable by the DAD system. Their unique chemical structures, as depicted in Figure 3, further accentuate their significance in the pharmacological spectrum of SM.

**Figure 4:**
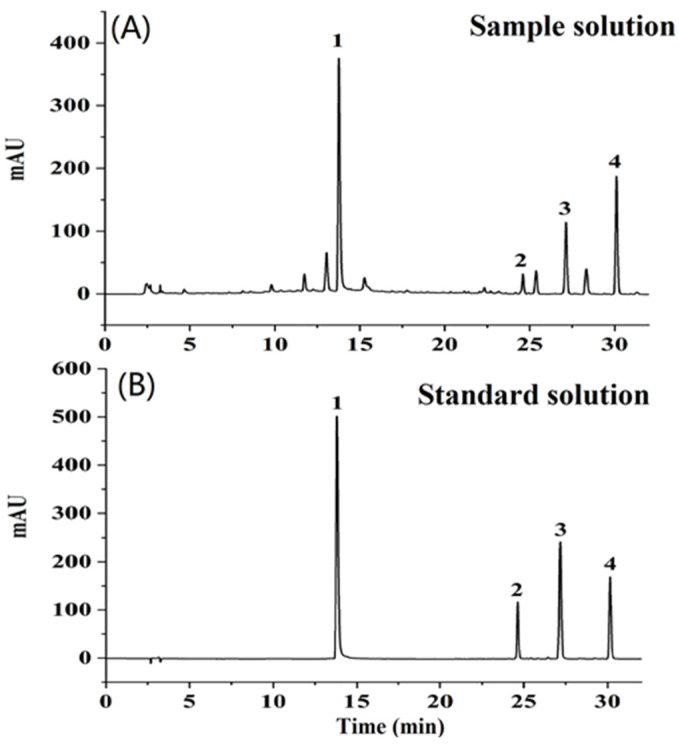
Representative HPLC chromatograms of sample solution (**A**) and standard solution (**B**).

Rigorous analysis of all 187 samples was undertaken and, for illustrative purposes, a representative HPLC chromatogram is exhibited in Figure 4.

This illustration clearly shows that the quartet of active constituents achieved baseline separation, thereby enabling their accurate quantification. Prior to the exhaustive testing of the SM samples, the robustness and reliability of the HPLC method were subjected to meticulous validation. Further insights into the interconnectedness of the five analyzed attributes were garnered through Pearson correlation analysis, and the derived coefficients were systematically recorded in Table S2. Notably, the most prominent correlation, with a coefficient of 0.64, was discerned between the concentrations of cryptotanshinone and tanshinone IIA, while other quality attributes displayed negligible correlations.

Detailed linearity data, as outlined in Table 1, reveal that the r^2^ values for the linearity equations corresponding to salvianolic acid B, dihydrotanshinone I, cryptotanshinone, and tanshinone IIA were impeccably close to 1, with values of 0.9998, 1.000, 1.000, and 1.000, respectively. Delving deeper into the method’s precision, Table 2 indicates that the intra-day and inter-day variations of the HPLC-DAD procedure were limited to 0.84% and 0.97%, respectively. The repeatability of the method, gauged by the relative standard deviation (RSD), was less than 0.83%. Recovery rates, a crucial metric for method validation, oscillated between 96.1% and 101.6%. Collectively, these metrics stand testament to the HPLC method’s superior sensitivity and accuracy, making it an exemplary tool for the quantitative determination of the quartet of active ingredients in SM.

Detailed linearity data, as outlined in Table 1, reveal that the r^2^ values for the linearity equations corresponding to salvianolic acid B, dihydrotanshinone I, cryptotanshinone, and tanshinone IIA were impeccably close to 1, with values of 0.9998, 1.000, 1.000, and 1.000, respectively. Delving deeper into the method’s precision, Table 2 indicates that the intra-day and inter-day variations of the HPLC-DAD procedure were limited to 0.84% and 0.97%, respectively. The repeatability of the method, gauged by the relative standard deviation (RSD), was less than 0.83%. Recovery rates, a crucial metric for method validation, oscillated between 96.1% and 101.6%. Collectively, these metrics stand testament to the HPLC method’s superior sensitivity and accuracy, making it an exemplary tool for the quantitative determination of the quartet of active ingredients in SM.

### 3.2. Measurement of Moisture Content

Before undertaking a hyperspectral quantitative analysis for the moisture content of SM, it is imperative to establish a dependable reference method. Moisture determination for all 187 batches of SM samples was conducted utilizing a rapid moisture analyzer. The obtained results elucidated that the moisture content within the SM samples ranged between 5.7% and 8.5%.

### 3.3. Division of Training Sets and Test Sets

During systematic evaluation, the 187 SM samples were stratified into training (calibration) sets and test sets employing the Kennard–Stone algorithm, maintaining a ratio of 4:1. Within this framework, the training sets were composed of 149 samples, while the test sets comprised the subsequent 38 samples to validate the proposed model. Table 3 delineates the content ranges for both the training (calibration) and test sets pertaining to the five analytes under investigation. It is noteworthy that the content distribution across both data sets exhibited uniformity, thereby facilitating the development of a model characterized by stability and robustness.

### 3.4. Performance of PLSR Model

In the realm of hyperspectral data analysis, preprocessing is often deemed an indispensable step prior to PLSR model development. However, upon meticulous evaluation of various preprocessing techniques, this study primarily resorted to the first derivative coupled with Savitzky–Golay smoothing methods. Astonishingly, the modeling outcomes derived from unprocessed raw data exhibited superior predictive capacities. Furthermore, when juxtaposing the outcomes of the successive projections algorithm and the uninformative variable elimination algorithm, the spectral bands delineated by the CARS algorithm proved to be more efficacious for modeling. A comprehensive display of the performance metrics of PLSR models integrated with diverse preprocessing techniques and band selection methodologies is provided in Table S3.

The model formulated utilizing the raw data, as filtered by the CARS algorithm, displayed the paramount $R_{c}^{2}$ and $R_{cv}^{2}$ values. Specifically, the $R_{c}^{2}$ and $R_{cv}^{2}$ values for salvianolic acid B, dihydrotanshinone I, cryptotanshinone, tanshinone IIA, and moisture content were discerned to be 0.281, 0.365, 0.026, 0.004, 0.009, 0.029, 0.019, 0.024, and 0.449, 0.672, in respective order. Moreover, the corresponding RPD metrics for these quality attributes within the PLSR framework were documented to be 1.254, 1.002, 1.015, 1.012, and 1.746, each of which was discernibly less than 2. Simultaneously, the RER values associated with these attributes were established to be −11.801, −0.107, 9.944, −1.494, and −0.031, respectively. These statistics unambiguously corroborate the limited predictive acumen of the PLSR model in this specific context.

### 3.5. Performance of SVMR and RBFNN Models

In Table S4, we present the analytical outcomes from both the support vector machine regression model (SVMR) and the radial basis function neural networks model (RBFNN). The $R_{c}^{2}$ and $R_{p}^{2}$ values for the quantification of salvianolic acid B, dihydrotanshinone I, cryptotanshinone, tanshinone IIA, and moisture content were observed to be suboptimal. Figures S1 and S2 depict the correlation plots contrasting the predicted outcomes from both SVMR and RBFNN with the experimentally determined values. Upon inspection, a discernible correlation between the modeled predictions and the empirical measurements appears to be absent.

### 3.6. Performance of SE-ResNet Model

The predictive efficacy of the refined SE-ResNet calibration model is delineated in Table 4. To provide a lucid comparative analysis between the algorithms, only the optimal results of the PLSR model are tabulated. The $R_{c}^{2}$ values for salvianolic acid B, dihydrotanshinone I, cryptotanshinone, tanshinone IIA, and moisture content were discerned to be 0.981, 0.980, 0.975, 0.972, and 0.970, respectively, while the $R_{cv}^{2}$ values were observed to be 0.975, 0.943, 0.962, 0.957, and 0.930, in respective order. Additionally, the RMSEP values for these components were ascertained to be 0.017, 0.028, 0.019, 0.024, and 0.031, respectively. Concurrently, the RPD metrics for salvianolic acid B, dihydrotanshinone I, cryptotanshinone, tanshinone IIA, and moisture content within the SE-ResNet framework were documented as 6.324, 4.188, 5.130, 4.822, and 3.780, respectively. Furthermore, RER values associated with these five quality parameters of the SE-ResNet model stood at 108.294, 2.250, 9.421, 5.292, and 0.903, respectively. Both the RPD and RER metrics testify to the superlative predictive prowess of the SE-ResNet model. The synergistic integration of ResNets with SE-Nets fosters an augmented performance, facilitating the acquisition of more discerning features whilst simultaneously curtailing the parameters and computational demands.

**Figure 5:**
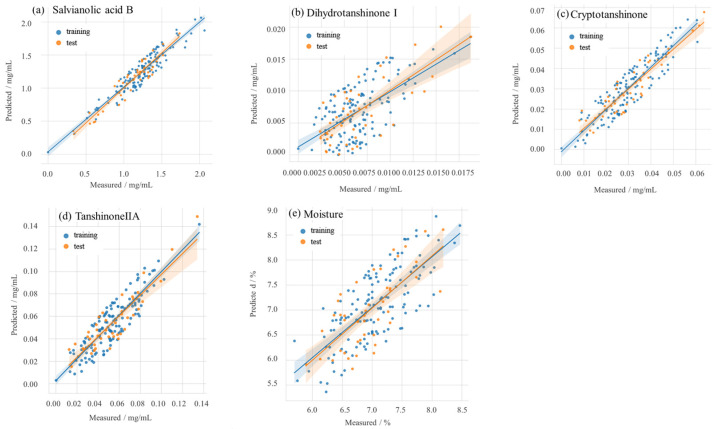
Correlation diagram of predicted values and measured values of bioactive compounds and moisture content.

The correlation plots juxtaposing the predictions rendered by the SE-ResNet model against the empirical measurements are elucidated in Figure 5. Models demonstrating elevated $R_{c}^{2}$, $R_{cv}^{2}$, and $R_{p}^{2}$ values inherently possess commendable predictive capabilities. Remarkably, all these metrics for the SE-ResNet model surpassed the 0.93 threshold. This implies that the model not only manifests an impeccable fit but also boasts high fidelity in prediction, underscored by its pronounced correlation and minimized error magnitude.

## 4. Discussion

PLSR is a common machine learning algorithm. Before we used the HSI data of the sample for PLSR modeling, we first developed a mask, selected the region of interest, calculated the average data, and performed Savitzky–Golay smoothing and first-order derivative preprocessing operations. We attempted to establish the PLSR model with the preprocessed data. However, the PLSR model is not suitable for a non-linear data set. The recorded data set by the HSI system in the reflectance mode is non-bilinear. So, the recorded spectra should first be transformed into absorbance mode for further analysis. However, in the present study, the PLSR as a linear model was applied to model a non-bilinear data set. We consider this to be the reason why the PLSR models were so inaccurate.

Therefore, we established the SVMR model and RBFNN model, but the results were still not ideal. The performance of the SVMR and RBFNN models are displayed in Table S4. The correlation diagrams of the results predicted by the SVMR and RBFNN models and real measured values are shown in Figures S1 and S2.

Pearson correlation was conducted to analyze the correlation between the five attributes investigated. The correlation coefficient is displayed in Table S2. The highest correlation coefficient, 0.64, is achieved between the contents of cryptotanshinone and tanshinone IIA. The correlations between other quality attributes are very weak.

In the present investigation, a novel methodology termed squeeze-and-excitation residual network combined hyperspectral image analysis (SE-ReHIA) was introduced for the concurrent assessment of quality attributes intrinsic to bulk medicinal materials. Specifically, the concentrations of salvianolic acid B, dihydrotanshinone I, cryptotanshinone, tanshinone IIA, and moisture were concurrently ascertained in *Salvia miltiorrhiza* (SM). The constructed model exhibited commendable predictive capabilities, positioning SE-ReHIA as a robust contender to the conventionally employed, labor-intensive HPLC approach. The SE-ReHIA method is discernibly more time-efficient, ecologically considerate, and preserves sample integrity. Moreover, the inherent capacity of the HSI system for real-time assessment bolsters its relevance within the preliminary material vetting phase of pharmaceutical manufacturing. Such integrations could considerably uplift batch-to-batch consistency, fortifying the reliability and uniformity of pharmaceutical products. It is noteworthy to mention that, in our survey of the literature, this research marks the inaugural application of the SE-ReHIA technique in the quality determination of SM. Our findings underscore the potential of HSI as a swift diagnostic tool for the projection of active ingredient concentrations and moisture levels in SM. However, more samples should be incorporated into the model for its application to real scenarios. In the future, the data of new samples will be added and the model re-trained. Prospective studies could pivot towards dissecting compositional dynamics of SM throughout its processing life cycle and during extended storage, further refining the quality assurance paradigms for bulk medicinal materials.

## 5. Conclusions

Our work demonstrates that SE-ReHIA is a viable alternative to the cumbersome HPLC method. It is faster, more environmentally friendly, and non-destructive. The HSI system is a quality control method that enables on-line detection, making it highly applicable in the raw material screening production line of botanical drugs. Its implementation can greatly enhance the consistency of drug batches, ensuring the stability of botanical drugs.

## Figures and Tables

**Table 1:** Calibration curves, correlation coefficients, linearity ranges, LOD, and LOQ of the HPLC method.

Analytes	Calibration Curves	r^2^	Linear Ranges (μg/mL)	LOD (μg/mL)	LOQ (μg/mL)
Salvianolic acid B	y = 7860.2x − 600.99	0.9998	13.420–2680	4.0260	13.420
Dihydrotanshinone I	y = 30,582x − 2.6289	1.0000	0.218–30	0.0654	0.218
Cryptotanshinone	y = 22,343x − 6.7114	1.0000	0.350–125	0.1050	0.350
Tanshinone IIA	y = 17,460x + 3.8458	1.0000	0.437–240	0.1311	0.437

**Table 2:** Precision, repeatability, stability, and recovery of the HPLC method (n = 6).

Analytes	Precision		Repeatability (RSD%)	Stability (RSD%)	Recovery (%, Mean/RSD)		
	Intra-Day	Inter-Day			Low	Medium	High
Salvianolic acid B	0.56	0.97	0.83	1.40	100.277 (0.33)	97.7829 (1.83)	98.1958 (1.23)
Dihydrotanshinone I	0.54	0.48	0.62	0.22	98.3369 (1.35)	100.410 (1.42)	99.5800 (0.52)
Cryptotanshinone	0.52	0.32	0.54	0.49	99.2654 (1.42)	96.0864 (1.23)	99.5132 (1.21)
Tanshinone IIA	0.84	0.32	0.50	0.43	98.8544 (1.83)	100.417 (1.53)	101.561 (0.68)

**Table 3:** Content ranges of five investigated analytes in different data sets.

Analytes	Training Set			Test Set		
	Min	Max	Mean	Min	Max	Mean
Salvianolic acid B	0.223	2.064	1.231	0.333	1.733	1.118
Dihydrotanshinone I	0.001	0.064	0.007	0.002	0.014	0.006
Cryptotanshinone	0.005	0.184	0.031	0.008	0.048	0.027
Tanshinone IIA	0.009	0.136	0.053	0.012	0.083	0.049
Moisture content	0.057	0.085	0.071	0.059	0.082	0.069

**Table 4:** Comparison between the performance of the SE-ResNet and PLSR models.

Algorithms	Analytes	Calibration		Validation		RER	RPD
		$R_{c}^{2}$	RMSEC	$R_{p}^{2}$	RMSEP		
SE-ResNet	Salvianolic acid B	0.980	0.110	0.975	0.017	108.294	6.324
	Dihydrotanshinone I	0.980	0.013	0.943	0.028	2.250	4.188
	Cryptotanshinone	0.975	0.015	0.962	0.019	9.421	5.130
	Tanshinone IIA	0.972	0.018	0.957	0.024	5.292	4.822
	Moisture content	0.970	0.020	0.930	0.031	0.903	3.780
PLSR	Salvianolic acid B	0.281	0.137	0.365	−0.156	−11.801	1.254
	Dihydrotanshinone I	0.003	0.163	0.004	−0.591	−0.107	1.002
	Cryptotanshinone	0.009	0.014	0.029	0.018	9.944	1.015
	Tanshinone IIA	0.019	0.218	0.024	−0.085	−1.494	1.012
	Moisture content	0.449	0.318	0.672	−0.913	−0.031	1.746

## Data Availability

Data are contained within the article.

## Supplementary Materials

The following supporting information can be downloaded at: https://www.mdpi.com/article/10.3390/s23239345/s1, Figure S1: Correlation diagram of predicted values by SVMR model and measured values of bioactive compounds and moisture content; Figure S2: Correlation diagram of predicted values by RBFNN model and measured values of bioactive compounds and moisture content; Table S1: Sample list of 187 batches of *Salvia miltiorrhiza*; Table S2: Correlation coefficients between the five quality attributes; Table S3: The performance parameters of PLSR algorithms with different band selection methods; Table S4: The performance parameters of SVMR and RBFNN algorithms.
