# Supplementary material for: A Nondestructive Methodology for Determining Chemical Composition of Salvia miltiorrhiza via Hyperspectral Imaging Analysis and Squeeze-and-Excitation Residual Networks
Source: Sensors (Basel). 2023 Nov 23;23(23):9345. doi: 10.3390/s23239345 (PMC10708663; doi:10.3390/s23239345)
Supplement: Supplementary file 1 [file sensors-23-09345-s001.zip › sensors-2682859-supplementary.pdf]

Table S1. Sample list of 187 batches of *Salvia miltiorrhiza*

Sample No.	Batch No.	Collected area
1	2022030802	Sichuan Province
2	2022052207	Sichuan Province
3	2022052306	Sichuan Province
4	2022060110	Sichuan Province
5	20220719006	Shanxi province
6	20220719016	Shanxi province
7	20220719017	Shanxi province
8	20220719023	Shanxi province
9	20220719033	Shanxi province
10	20220719038	Shanxi province
11	20220719039	Shanxi province
12	20220719040	Shanxi province
13	20220719046	Shanxi province
14	20220719047	Shanxi province
15	20220719053	Shanxi province
16	20220719057	Shanxi province
17	20220719061	Shanxi province
18	20220719062	Shanxi province
19	20220719067	Shanxi province
20	20220719069	Shanxi province
21	20220719070	Shanxi province
22	20220719071	Shanxi province
23	20220719075	Shanxi province
24	20220719082	Shanxi province

25	20220719086	Shanxi province
26	20220719091	Shanxi province
27	20200101	Shandong province
28	20200201	Shandong province
29	20200301	Shandong province
30	20200401	Shandong province
31	20200501	Shandong province
32	20200601	Shandong province
33	20200801	Shandong province
34	20200901	Shandong province
35	20201001	Shandong province
36	20201101	Shandong province
37	20201201	Shandong province
38	20210101	Shandong province
39	20210201	Shandong province
40	20210301	Shandong province
41	20210501	Shandong province
42	20210701	Shandong province
43	20210801	Shandong province
44	20210901	Shandong province
45	20211001	Shandong province
46	20211101	Shandong province
47	20211201	Shandong province
48	20220101	Shandong province
49	20220201	Shandong province
50	20220301	Shandong province

51	20220401	Shandong province
52	20220501	Shandong province
53	2022051508	Shandong province
54	2022052009	Shandong province
55	20210601	Shandong province
56	20220601	Shandong province
57	20220701	Shandong province
58	20220719002	Shandong province
59	20220719007	Shandong province
60	20220719012	Shandong province
61	20220719013	Shandong province
62	20220719024	Shandong province
63	20220719027	Shandong province
64	20220719030	Shandong province
65	20220719031	Shandong province
66	20220719032	Shandong province
67	20220719051	Shandong province
68	20220719059	Shandong province
69	20220719060	Shandong province
70	20220719063	Shandong province
71	20220719064	Shandong province
72	20220719072	Shandong province
73	20220719073	Shandong province
74	20220719074	Shandong province
75	20220719080	Shandong province
76	20220719081	Shandong province

77	20220719084	Shandong province
78	20220719093	Shandong province
79	20220719094	Shandong province
80	20220719099	Shandong province
81	210401	Shandong province
82	210501	Shandong province
83	210601	Shandong province
84	210701	Shandong province
85	210901	Shandong province
86	211001	Shandong province
87	211101	Shandong province
88	211201	Shandong province
89	220101	Shandong province
90	220301	Shandong province
91	220401	Shandong province
92	220501	Shandong province
93	220601	Shandong province
94	220701	Shandong province
95	2022041316	Chiatai Qingchunbao Pharmaceutical Co.
96	2022041619	Chiatai Qingchunbao Pharmaceutical Co.
97	2022041718	Chiatai Qingchunbao Pharmaceutical Co.
98	2022042114	Chiatai Qingchunbao Pharmaceutical Co.

99	2022042212	Chiatai Qingchunbao Pharmaceutical Co.
100	2022042403	Chiatai Qingchunbao Pharmaceutical Co.
101	2022042517	Chiatai Qingchunbao Pharmaceutical Co.
102	2022050115	Chiatai Qingchunbao Pharmaceutical Co.
103	2022040403	Anhui province
104	20220719001	Anhui province
105	20220719003	Anhui province
106	20220719004	Anhui province
107	20220719005	Anhui province
108	20220719011	Anhui province
109	20220719015	Anhui province
110	20220719019	Anhui province
111	20220719020	Anhui province
112	20220719021	Anhui province
113	20220719022	Anhui province
114	20220719028	Anhui province
115	20220719029	Anhui province
116	20220719048	Anhui province
117	20220719049	Anhui province
118	20220719078	Anhui province
119	20220719079	Anhui province
120	20220719085	Anhui province

121	20220719087	Anhui province
122	20220719088	Anhui province
123	20220719089	Anhui province
124	20220719090	Anhui province
125	20220719097	Anhui province
126	20200102	Henan province
127	20200202	Henan province
128	20200402	Henan province
129	20200502	Henan province
130	20200602	Henan province
131	20200702	Henan province
132	20200902	Henan province
133	20201102	Henan province
134	20201202	Henan province
135	20210102	Henan province
136	20210202	Henan province
137	20210402	Henan province
138	20210502	Henan province
139	20210602	Henan province
140	20210802	Henan province
141	20210902	Henan province
142	20211002	Henan province
143	20211102	Henan province
144	20211202	Henan province
145	20220102	Henan province
146	20220202	Henan province

147	20220301	Henan province
148	20220302	Henan province
149	20220307	Henan province
150	20220502	Henan province
151	20220602	Henan province
152	20220702	Henan province
153	20220719009	Henan province
154	20220719010	Henan province
155	20220719014	Henan province
156	20220719025	Henan province
157	20220719026	Henan province
158	20220719034	Henan province
159	20220719035	Henan province
160	20220719036	Henan province
161	20220719037	Henan province
162	20220719041	Henan province
163	20220719042	Henan province
164	20220719043	Henan province
165	20220719044	Henan province
166	20220719045	Henan province
167	20220719050	Henan province
168	20220719052	Henan province
169	20220719054	Henan province
170	20220719055	Henan province
171	20220719056	Henan province
172	20220719058	Henan province

173	20220719065	Henan province
174	20220719066	Henan province
175	20220719068	Henan province
176	20220719076	Henan province
177	20220719077	Henan province
178	20220719083	Henan province
179	20220719092	Henan province
180	20220719095	Henan province
181	20220719096	Henan province
182	20220719098	Henan province
183	20220719100	Henan province
184	2022050111	Yunnan province
185	2022051505	Yunnan province
186	2022052504	Yunnan province
187	2022070101	Yunnan province

Table S2. Correlation coefficients between the five quality attributes

	Salvianolic Acid B	Dihydrotanshinone I	Cryptotanshinone	Tanshinone IIA	moisture
Salvianolic Acid B	1	—	—	—	—
Dihydrotanshinone I	-0.081	1	—	—	—
Cryptotanshinone	0.072	0.354	1	—	—
Tanshinone IIA	0.094	0.320	0.640	1	—
moisture	0.148	-0.053	0.018	0.035	1

Table S3. The performance parameters of PLSR algorithms with different band selection methods

Band selection method	Analysis	Pretreatment method	Calibration		Cross-validation		Validation		Selected wavelengths number
			R_C^2	RMSEC	R_{cv}^2	RMSECV	R_p^2	RMSEP	
CARS	Salvianolic acid B	Raw data	0.2810	0.1374	0.3758	-1.1507	0.3648	-0.1570	7
		First derivative	0.2925	0.0648	0.3239	-0.5977	0.3674	-0.1739	7
		SG smooth	0.2916	0.0709	0.3037	-0.4041	0.3590	-0.1209	7
	Dihydrotanshinone I	Raw data	0.0026	0.1626	0.0114	-0.0062	0.0040	-0.5910	4
		First derivative	0.0026	0.1874	0.0115	-0.0363	0.0042	-0.8107	4
		SG smooth	0.0028	0.0030	0.0117	-0.0689	0.0032	-0.0195	4
	Cryptotanshinone	Raw data	0.0102	0.1911	0.0134	-0.0967	0.0300	-0.0354	22
		First derivative	0.0096	0.2806	0.0152	-0.4167	0.0296	-0.0086	22
		SG smooth	0.0105	0.1347	0.0129	-0.0239	0.0293	0.0131	22
	Tanshinone IIA	Raw data	0.0193	0.2178	0.0249	-0.1043	0.0235	-0.0853	9
		First derivative	0.0201	0.1481	0.0256	-0.1670	0.0235	-0.0848	9
		SG smooth	0.0204	0.1190	0.0243	-0.0501	0.0237	-0.1041	9
	Moisture content	Raw data	0.4846	0.2044	0.5212	-0.1894	0.5581	-0.3195	22
		First derivative	0.4829	0.2100	0.5386	-0.2705	0.5543	-0.3020	22
		SG smooth	0.5202	0.0832	0.5495	-0.3221	0.5131	-0.1157	22

SPA	Salvianolic acid B	Raw data	0.2944	0.0529	0.2887	-0.2690	0.3210	0.1040	2
		First derivative	0.2999	0.0171	0.2790	-0.1856	0.3425	-0.0198	2
		SG smooth	0.2995	0.0196	0.2894	-0.2758	0.3289	0.0597	2
	Dihydrotanshinone I	Raw data	0.0028	0.0232	0.0117	-0.0701	0.0031	0.0268	2
		First derivative	0.0028	0.0181	0.0117	-0.0654	0.0031	0.0033	2
		SG smooth	0.0028	0.0034	0.0117	-0.0720	0.0032	-0.0199	2
	Cryptotanshinone	Raw data	0.0112	0.0164	0.0130	-0.0393	0.0297	-0.0122	22
		First derivative	0.0111	0.0337	0.0136	-0.1217	0.0297	-0.0148	22
		SG smooth	0.0113	0.0017	0.0129	-0.0090	0.0301	-0.0426	22
	Tanshinone IIA	Raw data	0.0217	0.0060	0.0238	-0.0070	0.0227	-0.0082	2
		First derivative	0.0210	0.0657	0.0247	-0.0804	0.0227	-0.0117	2
		SG smooth	0.0216	0.0137	0.0241	-0.0274	0.0231	-0.0473	2
	Moisture content	Raw data	0.5398	0.0130	0.4946	-0.0710	0.4617	0.0968	2
		First derivative	0.5404	0.0105	0.4931	-0.0648	0.4822	0.0151	2
		SG smooth	0.5395	0.0141	0.4856	-0.0324	0.4734	0.0505	2

UVE	Salvianolic acid B	Raw data	0.3021	0.0029	0.2570	-0.0055	0.3447	-0.0330	1
		First derivative	0.3016	0.0059	0.2604	-0.0323	0.3319	0.0421	1
		SG smooth	0.3025	0.0001	0.2593	-0.0240	0.3413	-0.0257	1
	Dihydrotanshinone I	Raw data	0.0028	0.0310	0.0117	-0.0597	0.0033	-0.1042	1
		First derivative	0.0027	0.0591	0.0116	-0.0434	0.0034	-0.1820	1
		SG smooth	0.0028	0.0016	0.0117	-0.0715	0.0032	-0.0246	1
	Cryptotanshinone	Raw data	0.0094	0.3113	0.0124	0.0547	0.0297	-0.0150	48
		First derivative	0.0088	0.3887	0.0143	-0.2416	0.0292	0.0186	48
		SG smooth	0.0105	0.1306	0.0126	0.0274	0.0294	0.0074	48
	Tanshinone IIA	Raw data	0.0187	0.2586	0.0233	0.0397	0.0248	-0.2121	10
		First derivative	0.0197	0.1805	0.0239	-0.0164	0.0246	-0.1833	10
		SG smooth	0.0204	0.1245	0.0250	-0.1090	0.0231	-0.0431	10
	Moisture content	Raw data	0.4488	0.3176	0.5980	-0.5661	0.6719	-0.9133	11
		First derivative	0.4930	0.1767	0.5569	-0.3580	0.5511	-0.2869	11
		SG smooth	0.5184	0.0896	0.5260	-0.2113	0.5066	-0.0875	11

Table S4. The performance parameters of SVMR and RBFNN algorithms

Algorithms	Analytes	Training sets		Test sets	
		$R_C{}^2$	RMSEC	$R_p{}^2$	RMSEP
SVMR	Salvianolic acid B	0.47	0.23	0.33	0.19
	Dihydrotanshinone I	-18.72	0.03	-87.36	0.03
	Cryptotanshinone	-0.67	0.03	-0.17	0.02
	Tanshinone IIA	-12.21	0.06	-20.37	0.06
	Moisture content	0.10	0.49	-0.12	0.56
RBFNN	Salvianolic acid B	0.41	0.25	0.17	0.22
	Dihydrotanshinone I	-45496.87	1.23	-193176.32	1.18
	Cryptotanshinone	0.06	0.02	-0.07	0.01
	Tanshinone IIA	0.14	0.02	0.04	0.02
	Moisture content	-0.18	0.57	-0.16	0.57

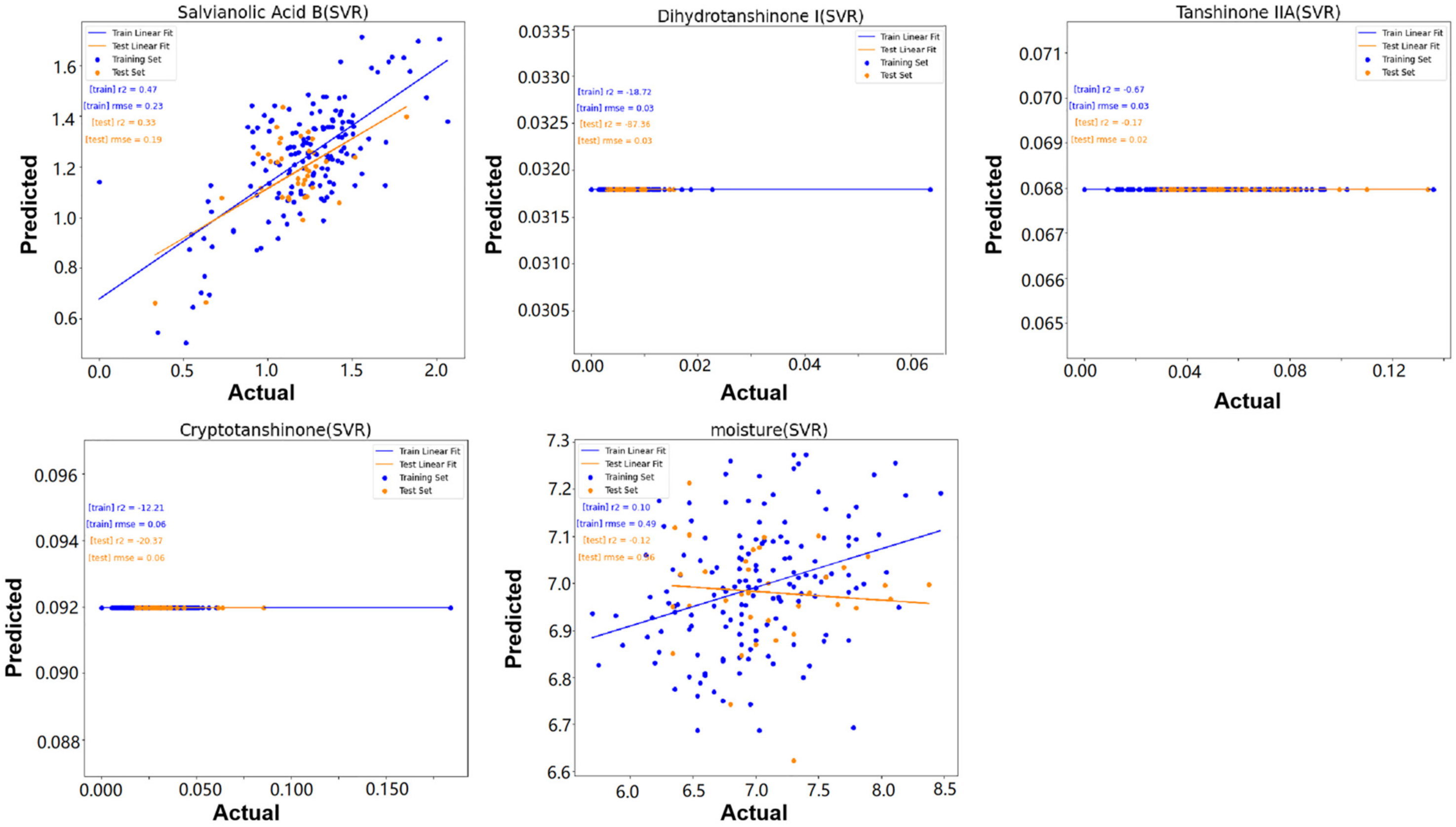

Figure S1. Correlation diagram of predicted values by SVMR model and measured values of bioactive compounds and moisture content

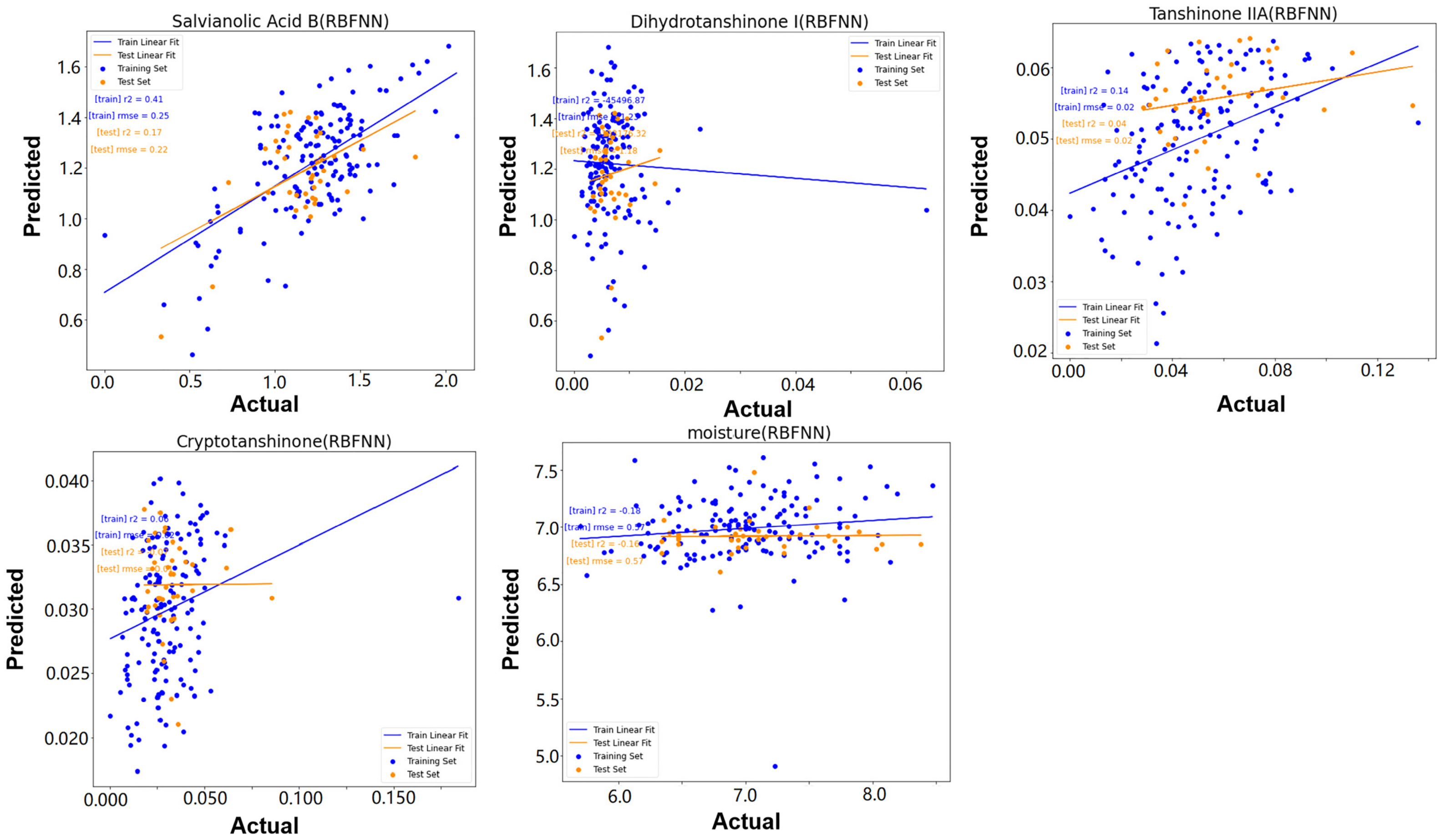

Figure S2. Correlation diagram of predicted values by RBFNN model and measured values of bioactive compounds and moisture content
